# Supporting underserved patients with their medicines: a study protocol for a patient/professional coproduced education intervention for community pharmacy staff to improve the provision and delivery of Medicine Use Reviews (MURs)

**DOI:** 10.1136/bmjopen-2016-013500

**Published:** 2016-12-09

**Authors:** Asam Latif, Kristian Pollock, Claire Anderson, Justin Waring, Josie Solomon, Li-Chia Chen, Emma Anderson, Sulma Gulzar, Nasa Abbasi, Heather Wharrad

**Affiliations:** 1 Faculty of Medicine and Health Sciences, School of Health Sciences, University of Nottingham, Nottingham, UK; 2 Division of Social Research in Medicines and Health, School of Pharmacy, University of Nottingham, Nottingham, UK; 3 Centre for Health Innovation, Leadership and Learning, University of Nottingham, Nottingham, UK; 4 School of Health and Life Sciences, Leicester School of Pharmacy, De Montfort University, Leicester, UK; 5 Centre for Pharmacy Postgraduate Education (CPPE), University of Manchester, Manchester, UK; 6 NHS South East Staffordshire and Seisdon Peninsular CCG, Burton on Trent, Staffordshire Nottingham, Staffordshire, UK; 7 The Queen's Pharmacy Centre, Nottingham, UK

**Keywords:** EDUCATION & TRAINING (see Medical Education & Training), PRIMARY CARE

## Abstract

**Introduction:**

Community pharmacy increasingly features in global strategies to modernise the delivery of primary healthcare. Medicine Use Reviews (MURs) form part of the English Government's medicines management strategy to improve adherence and reduce medicine waste. MURs provide space for patient–pharmacist dialogue to discuss the well-known problems patients experience with medicine taking. However, ‘underserved’ communities (eg, black and minority ethnic communities, people with mental illness), who may benefit the most, may not receive this support. This study aims to develop, implement and evaluate an e-learning education intervention which is coproduced between patients from underserved communities and pharmacy teams to improve MUR provision.

**Methods and analysis:**

This mixed-methods evaluative study will involve a 2-stage design. Stage 1 involves coproduction of an e-learning resource through mixed patient–professional development (n=2) and review (n=2) workshops, alongside informative semistructured interviews with patients (n=10) and pharmacy staff (n=10). Stage 2 involves the implementation and evaluation of the intervention with community pharmacy staff within all community pharmacies within the Nottinghamshire geographical area (n=237). Online questionnaires will be completed at baseline and postintervention (3 months) to assess changes in engagement with underserved communities and changes in self-reported attitudes and behaviour. To triangulate findings, 10 pharmacies will record at baseline and postintervention, details of actual numbers of MURs performed and the proportion that are from underserved communities. Descriptive and inferential statistics will be used to analyse the data. The evaluation will also include a thematic analysis of one-to-one interviews with pharmacy teams to explore the impact on clinical practice (n=20). Interviews with patients belonging to underserved communities, and who received an MUR, will also be conducted (n=20).

**Ethics and dissemination:**

The study has received ethical approval from the NHS Research Ethics Committee (East Midlands–Derby) and governance clearance through the NHS Health Research Authority. Following the evaluation, the educational intervention will be freely accessible online.

**Strengths and limitations of this study**

- The involvement of underserved communities in the e-learning development process is central to the coproduction model. This provides a platform for their voices to be heard. It also incorporates the views of community pharmacy staff to ensure the intervention is sensitive to the context in which pharmacy teams deliver Medicine Use Reviews (MURs).
- There is a risk of social desirability bias through the implementation of self-reported questionnaires. We will seek to minimise this using self-completion online questionnaires and not through face-to-face completion.
- To triangulate findings, self-reported pharmacy staff behaviour change will be compared with the numbers of MURs performed with patients from underserved communities at baseline and postintervention. Pharmacy staff and patient interview accounts will further inform the interpretation of questionnaire findings and any impact on practice.

## Introduction

Globally, community pharmacy increasingly features in delivering new services to modernise primary healthcare, often in response to the growing demand on general practitioners (GPs) and the shifting of care from resource impoverished hospital settings to comparatively cheaper care in the community.^1^ ^2^ In England, one area that has seen significant investment is pharmacy medicine management services.^3^ This is due to increasing recognition that patients experience widespread problems taking medicines and have concerns about dependence, tolerance and side effects that affect medicine adherence.^4^ How well people understand the necessity to take their medicine(s) and how well supported (practically and psychologically) they are to do so will also affect medicine use. One independent report evaluating the scale, causes and costs of wasted medicines estimates that in England alone, the annual cost of NHS prescription medicines wastage is £300 million, including £90 million worth of unused prescription medicines that are retained in individuals' homes and £110 million returned to community pharmacies.^5^

To support medicine taking and to tackle problems of non-adherence and medicine waste, community pharmacies are increasingly being commissioned to deliver new services to support patient medicine taking.^6^ Since 2005, the NHS Medicine Use Review (MUR) service has been available from English community pharmacies. MURs aim to support patients' understanding and adherence to therapy and to reduce avoidable waste.^7^ They involve a patient–pharmacist face-to-face discussion, to establish a picture of the patient's medicine use and resolve medicine problems or concerns that the patient may be experiencing. Pharmacists must be accredited through additional training before they offer the service. Pharmacies claim £28 from the NHS for each MUR undertaken and can undertake a maximum of 400 MURs in a financial year; meaning an annual total not exceeding £11 200 per pharmacy is paid by the NHS.

Despite over 3 million MURs being conducted in 2015–2016,^7^ there have been questions over their value to improve medicine use.^8^ ^9^ The former Primary Care Trusts have indicated they were not being targeted to ‘*local needs and patient priorities*’.^10^ Commercial and work pressures have resulted in a ‘quantity driven’ approach whereby pharmacists invite patients on simpler medicine regimes that can be performed quickly, over those with complex needs.^11–13^ Commissioners responded to these concerns in 2011 and 2014, with reforms stipulating that at least 70% of all MURs undertaken should be with patients in higher risk groups (high-risk medicines, respiratory and cardiovascular disease and people recently discharged from hospital).

However, importantly, there is no obligation to target patients from underserved or ‘seldom heard voice’ communities (eg, people with disability, people who are housebound, people from the black and minority ethnic community, people with mental illness). Although patients from these communities will have unique needs, they are all less inclined to participate in health or screening services and have poorer health outcomes.^14^ There is increasing evidence, suggesting that shared decision-making interventions significantly improve outcomes for disadvantaged patients and may be more beneficial to these groups than higher literacy/socioeconomic status patients.^14^ They may therefore benefit most from the MUR service. With over 90% of all pharmacies in England now providing MURs, there is a strong need for an educational intervention to improve pharmacists' attitudes and behaviour towards underserved groups, so they are not further marginalised from receiving MURs.

The coproduction philosophy, where health professionals and service users work in partnership to improve the patients' experience, is increasingly being used to improve services to patients.^15^ ^16^ The coproduction concept is broad and can range from service coplanning and cocommissioning, service codesign and codelivery, through to service coassessment, comonitoring and coevaluation.^17^ Central to this idea is the contribution of service users that allow services to be tailored while also acknowledging the contribution from front-line healthcare staff.^18^ Such an educational intervention is uniquely placed to address the clearly defined knowledge gaps (eg, diversity awareness, unique needs of underserved communities, cultural competence issues) present in the case of MUR provision among pharmacists.

An e-learning intervention was identified as the most appropriate and cost-efficient method to deliver the training. E-learning tools have a track record in educating health professionals and are improving health-related behaviours.^19^ ^20^ They have also been identified by the NHS national learning strategy as a delivery mechanism to ensure training is relevant and flexible enough to take into account different learning styles^21^ as well as being an effective and flexible way to deliver health professional training.^22^ In this paper, we present the protocol for developing and evaluating the coproduced e-learning educational intervention.

### Aims and objectives

This study aims to investigate whether an educational intervention coproduced with patients and professionals can change pharmacy staff attitudes and behaviour to improve the provision of MURs to underserved communities. The primary objectives are to:

1. Coproduce, with patients from underserved communities and pharmacy teams, an e-learning resource designed to change the attitudes and behaviour of pharmacy staff to improve the provision of MURs to underserved communities.
2. Evaluate the impact of the e-learning resource on pharmacy staff's reported behaviour scores on providing MURs to underserved communities.
3. Characterise pharmacy staff's experience, perceived impact on practice and to investigate barriers and facilitators to successful implementation of the e-learning.

The secondary objectives are to:

1. Explore underserved patient experiences of medicines and levels of support received from healthcare professionals.
2. Investigate patients' experiences of community pharmacy service, their feelings of being offered and undertaking an MUR and how this has affected their knowledge of medicines and their use and perceptions of pharmacy services.

## Methods and analysis

**Figure 1:**
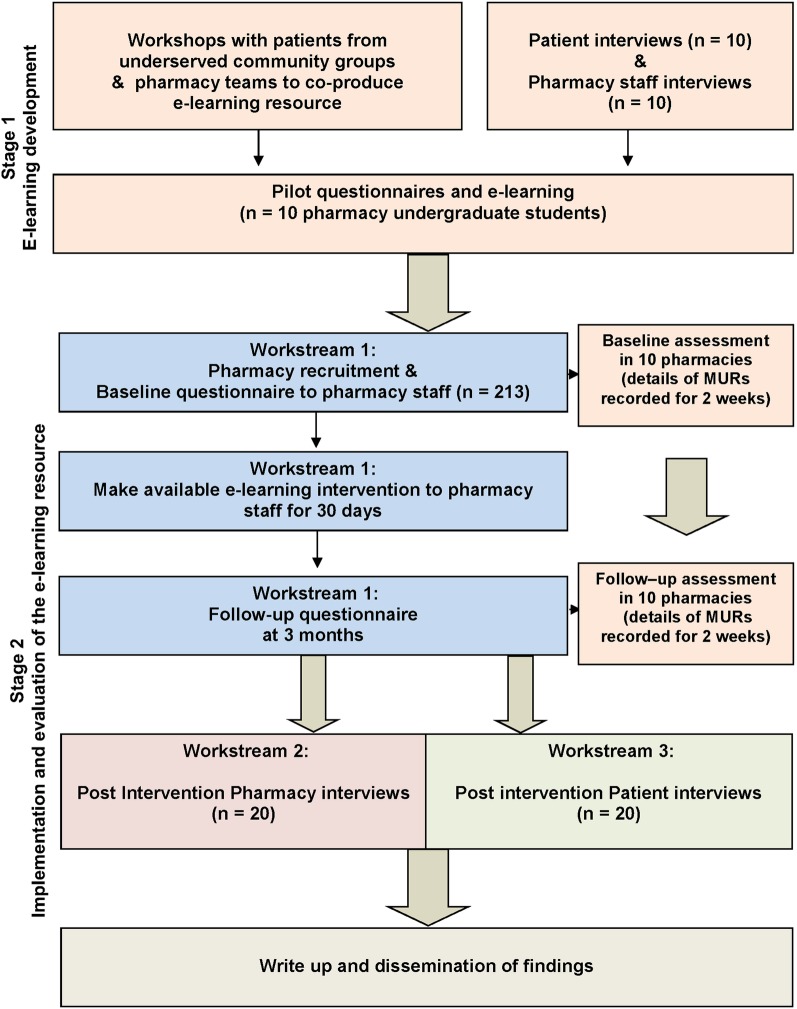
Study flow diagram.

This study will be conducted in two stages (figure 1) at multiple pharmacies. Multiple sources of data will be collected to contextualise and converge lines of inquiry, including prequestionnaire and postquestionnaire surveys and qualitative interviews with patients and pharmacy staff.

### Description of e-learning educational intervention

The pharmacist e-learning educational intervention will take the form of a series (2–4) of reusable learning objects (RLOs). RLOs are small, pedagogically designed, ‘bite-sized’ chunks of e-learning that focus on a particular topic.^22^ The contents, including visual illustrations and interactivities, will originate from mixed patient–professional development workshops. These workshops are designed to capture patient and pharmacy team experiences and ideas on how staff can better engage with patients from underserved communities.

### Sample and recruitment

#### Stage 1: coproduce e-learning resource

Pharmacy teams and patient groups from underserved communities will be recruited to workshops to coproduce the e-learning resources to inform the intervention. ‘Pharmacy teams’ will include participants from the following groups (criteria outlined in box 1) who will be sampled and recruited purposefully (n=15 participants).

- Community pharmacy staff (pharmacists, accredited technicians, dispensers, medicine counter assistants).
- Superintendent pharmacists, community pharmacy owners.
- Pharmacy representatives/professional leadership bodies/educational bodies (ie, Nottingham Local Pharmaceutical Committee (LPC), Royal Pharmaceutical Society, Pharmacy Voice, Centre for Pharmacy Postgraduate Education (CPPE)). EA is a CPPE tutor who has experience running pharmacy education workshops and will facilitate recruitment.

Box 1 **Eligibility criteria for the selection of pharmacy teams**

Inclusion criteria

1. Community pharmacy staff who have active involvement (on a day-to-day basis) in identifying, inviting or undertaking Medicine Use Reviews (MURs) within a community pharmacy

2. Willing to provide consent to take part in the workshop activities/interview

Exclusion criteria

1. Pharmacy staff who are not actively involved with identifying, inviting or undertaking MURs within a community pharmacy

2. Unwilling to provide consent to take part in the workshop activities/interview

Patients from underserved communities will be recruited via local organisations (n=15 participants) using the eligibility criteria detailed in box 2. These groups have been identified from the previous literature into the MUR service and more recent work on the New Medicines Service (NMS) evaluation study.^23^ Healthwatch Nottingham (part of Healthwatch England), along with other organisations that represent underserved communities, will be approached and will facilitate patient recruitment.

Box 2 **Eligibility criteria for the selection of underserved patients for reusable learning object development workshop**

Inclusion criteria

1. Patients (or organisational representative) identified as belonging to (or representing) an underserved community. For the purposes of this study, the term ‘underserved’ is used to describe patients who are eligible to receive the Medicines Use Review (MUR) service, but who, for whatever reason, not receive the service. We define patients from ‘underserved’ communities as including, but not limited to people with:

- People whose first language is not English
- People with physical, visual, hearing or learning impairments, or any other disability
- Patients taking multiple medicines for more than one illness or condition
- People who are housebound or their carers
- Black and minority ethnic communities
- People with mental illness
- People who are homeless or have no fixed address
- People from refugee, asylum and traveller communities
- People from Lesbian, Gay, Bisexual and Transgender communities
- Men aged 16–25

2. Able to understand study information, willing and have capacity to consent to take part in the workshops/interview

Exclusion criteria

1. Not belonging to (or representing) an underserved community

2. Unable to understand study information, unwilling or unable to provide informed consent to the workshops/interview (translational services will be available should participants be unable to read and converse in English)

In addition, one-to-one interviews will be undertaken with representatives from pharmacy teams (n=10) and patients from underserved communities (n=10) to further inform the e-learning intervention. The inclusion/exclusion criteria will be as above (boxes 1 and 2).

#### Patient and public involvement

Two patient and public involvement (PPI) representatives from undeserved communities, who are eligible themselves for an MUR, will be recruited to form an advisory panel for the study. PPI representatives will be recruited via the East Midlands Academic Health Science Network (AHSN) ‘Public Face’ newsletter. The AHSN was established by NHS England in 2013 to improve health and social care by bringing patients, carers and communities together with health and social care providers, industry and researchers.

The PPI advisory panel will meet regularly to advise and challenge where necessary on the study design and information included in the e-learning educational intervention. Their involvement in the project will include attending research meetings, workshops, providing feedback on the development of the RLOs and study findings. They will bring their experience and expertise of the world outside academia and their time living with a long-term condition to ensure the patient voice is heard.

#### Stage 2: implementation and evaluation of the e-learning educational intervention

The implementation and evaluation of the e-learning intervention will be assessed through three work streams.

#### Work stream 1: pre–postquestionnaire survey

All community pharmacies (n=237) in Nottinghamshire will be approached to take part in the study. An information sheet and invitation letter will be sent to pharmacies which are currently providing the MUR service to patients. Frequently, pharmacy support staff are actively involved (on a day-to-day basis) in identifying and inviting patients to the MUR service. Pharmacists and their support staff will therefore be invited to participate in this study. The study is not powered to detect differences as there is no prior study on which to base a power calculation. It is assumed that 90% of the pharmacies in Nottinghamshire (n=237) are offering the MUR. With an estimated one pharmacist and one support-staff expressing interest to be involved, a response rate of 50% will give a minimum sample of 213 participants.

#### Work stream 2: qualitative appraisal of the educational intervention: pharmacy staff interviews

Following completion of the postintervention questionnaire, a sample of pharmacists and support staff (n=20) who have completed the online questionnaire will be invited to take part in a semistructured interview to further explore their experience of using the e-learning educational resource and its impact on practice.

#### Work stream 3: qualitative appraisal of the MUR: patient interviews

Following their MUR, a purposive sample of patients from underserved communities that have had an MUR, will be provided with information about the study by the pharmacist. If the patient agrees to take part, a one-to-one, face-to-face or telephone interview (n=20) will be arranged. Interviews will explore their overall perceptions of MURs, in particular, their experience of how they were approached and engaged.

### Study procedures

This study will involve two stages that will be undertaken between August 2016 and March 2019 (figure 1).

#### Stage 1: workshops and interviews to coproduce e-learning resource

The purpose of the workshops will be to help codevelop an e-learning training package for pharmacists to improve the provision and number of MURs to underserved communities. Initial development workshops (n=2) will focus on capturing themes through stories and experiences, including design aspects such as preferred media, on ‘storyboards’ to elicit the contents of the RLOs prior to production and enable an RLO specification to be written. The workshop will begin by providing participants with an outline of the day and reminder that there are no right/wrong answers. An ‘appreciative’ method will be used throughout the workshop to promote inclusivity. Examples of existing RLOs will be provided as well as an explanation of how they are developed. Small groups will work to explore (on flip-charts) what they think is important to include in a pharmacy staff e-learning intervention. Anticipated RLO topics may include: ‘identifying and learning about underserved communities’, ‘effective targeting and invitation strategies’ and ‘tailoring consultation skills to meet the needs of underserved communities’.

Following initial concept ideas gathered from the development workshops and interviews, an iterative e-learning development process will be conducted with two ‘review’ workshops with patients from underserved communities and pharmacy teams. These workshops will be identical to the development sessions, but will be used to further develop and refine the e-learning specification. This will enable the refinement of initial ideas into a detailed specification (working title, learning objectives, contents of topics covered) to be developed. Workshops will be held at the University of Nottingham and will be facilitated by AL (chief investigator (CI)), HW, other members of the University's HELM (Health and E-Learning and Media) team and PPI panel representatives will be present. A £200 inconvenience allowance will be made available to all participants.

A review of the literature will ensure the contents are factually correct. The specification will be peer reviewed by experts in the field for clarity, factual content and appropriateness of any animations. For example, the peer review may identify errors in the content, and/or suggest content changes to improve the e-learning. These comments will be fed back to the author for revision of the specification and improvement.

Software development and further peer review of the final RLOs will be undertaken before release.^22^ Errors or problems with functionality that are identified will require the e-learning to be reviewed by the developer. Such errors are typically minor and do not require the RLO to be further peer reviewed.

One-to-one semistructured interviews will be undertaken by the CI with patients and pharmacists to further inform the e-learning intervention. These interviews will take place at a time and place convenient to the participant and, with consent, will be audio recorded. Pharmacy staff will be invited to an interview to explore existing practice and experience of providing MURs to underserved groups and to identify knowledge gaps that will help create the contents for the e-learning materials. Patients from underserved communities who may be unable or unwilling to attend a workshop (ie, people who are housebound, traveller community) will be invited. These interviews will explore medicine use, support from other health professionals (eg, from the practice nurse), perceptions of the pharmacy and views on what would the patient like to see emphasised in an e-learning intervention.

#### Piloting of RLOs and evaluation tools

The e-learning resource and online questionnaires will be piloted on 10 undergraduate pharmacy students from the University of Nottingham to test the functionality of the RLOs and face validity of the data collection instruments. This will be performed by asking students to work through the e-learning and checking ease of use. Face validity of the questionnaire will also be assessed.

#### Stage 2: implementation and evaluation

Following permission from the pharmacy owner/manager, the pharmacist in charge of the pharmacy will be contacted and will inform their team and provide them with the study information. A poster will also be sent to each pharmacy to be displayed in staff areas to raise awareness. Assistance in recruiting pharmacies will be sought from LPCs, other pharmacy bodies and the NIHR Comprehensive Local Research Network (CLRN).

#### Implementation of e-learning and outcome measures

All pharmacy staff will be invited to complete an online, baseline questionnaire which will be accessible over 3 weeks. Following baseline data collection (with a reminder after 2 weeks), the online e-learning educational intervention will be made available to participants for 30 days. A postintervention questionnaire will then be sent at 3 months to those who completed the baseline questionnaire (with one reminder). Consent to take part will be implied through the completion of the questionnaires.

The questionnaire will collect the following data: (1) participant characteristics, that is, age, gender, educational achievement and details of any MUR training received, years qualified as a registered pharmacist; (2) pharmacy characteristics, for example, geographical area, ownership type (eg, large multiples, supermarkets) and economic deprivation (deprivation index will be obtained through the UK Office of National Statistics (ONS) data for each pharmacy using the postcode as the lookup reference); (3) pharmacist and support-staff attitudes will be assessed through attitudinal statements developed from the ‘inequalities imagination’ framework^24^ (this framework enables healthcare professionals to more fully meet the needs of ‘underserved’ or ‘disadvantaged’ patients) and (4) pharmacist/support-staff behaviour change assessed through a 12-item theory-based instrument.^25^ This validated tool with robust metric properties is used to assess the impact of continuing professional development activities on health professionals' clinical behavioural intention change.

Whereas self-reporting is a practical and efficient method of collecting data, there are limitations such as relying on respondents' honesty and accuracy of reporting. In order to triangulate and support questionnaire findings of self-reported behaviour with actual practice, ∼10 of the pharmacies taking part will be invited to record the number of MURs performed and how many were from underserved communities over a 2-week period. The sample size for the number of pharmacies included will be guided by the feasibility of data collection and resource constraints.

This will occur at baseline and at 3 months following the e-learning.

#### Work stream 2: pharmacy staff interview

A purposeful sample of community pharmacy staff will be invited to a one-to-one interview about their experience of the e-learning, its usability (barriers and facilitators) of the RLOs and its effect on daily practice. Written consent will be taken before the start of the interview and permission sought for the interview to be audio recorded. It will be explained to the potential participant that entry into the study is entirely voluntary. The interviews will last for ∼30 min and will be conducted at the place of work or at any other convenient location according to participant preferences.

#### Work stream 3: patient interviews

A purposeful sample of at least 10 community pharmacies will be recruited to help identify patients from underserved groups. The pharmacist will sequentially invite every eligible patient at the end of their MUR to see if they are interested in taking part in the study. If the patient expresses interest, the pharmacist will hand them a study information sheet and seek consent for their details to be passed onto the CI. The patient will then be contacted by phone by the CI and the study will be fully explained. They will then be given time to decide whether to take part. If they agree, they will be invited to interview. The purpose of the interview will be to explore how the patient felt about being approached and engaged for the MUR, whether the strategies that were included in the e-learning were used by pharmacy staff and what benefit the MUR had to improve medicine understanding and use.

### Analyses

#### Quantitative data analysis

Descriptive statistics will be used to describe participants' demographic and baseline characteristics. Continuous data will be presented using means and SDs if approximately normally distributed, and medians and IQR if non-normally distributed. Categorical data will be described using frequencies and percentages. To assess the effect of the e-learning, baseline and postintervention data scores will be compared. Categorical variables will be analysed using the χ^2^ test or Fisher's exact test as appropriate. Continuous data will be analysed using the within-group t-test or Wilcoxon signed rank-test as appropriate. Statistical significance will be assessed at the 5% (two-sided) level. All statistical analyses will be conducted using IBM Statistical Package for Social Sciences (SPSS) 22.

#### Qualitative data analysis

All interviews will be transcribed verbatim and the data imported into qualitative analysis package NVivo; QSR International Pty for the purpose of coding and thematic analysis.^26^ Data analysis will start during the early stages of data collection and proceed iteratively in order for emergent findings to be incorporated into subsequent data collection, including the revision of data collection tools, such as interview topic guides. A theoretical framework around diversity and inequities in health will guide qualitative data collection and analysis.^24^

Initial reading and rereading of the transcribed data will be undertaken, with feedback and checking by several members of the qualitative research team, to identify common codes and categories. Actively searching for disconfirming data will be undertaken as well as regular detailed discussions among the qualitative researchers and PPI advisory panel. Consideration will then be given to how these issues group together in broader themes related to the research objectives. The principle of constant comparison will be used to test and refine the empirical conceptual consistency of codes and themes, which have been synthesised and narrated.^27^

## Ethics and dissemination

The study will be conducted in accordance with the ethical principles that have their origin in the Declaration of Helsinki, 1996; the principles of Good Clinical Practice and the Department of Health Research Governance Framework for Health and Social Care, 2005. All participants will be informed that participation (in any stage of the study) will be voluntary and that they may withdraw at any time.

### Patient participation in workshops

Central to the development of the e-learning educational intervention are the views of patients from underserved communities. A coproduction methodology will be used to enable patients and professionals to work in partnership to codesign the pharmacy e-learning intervention. By working alongside each other, a better understanding of the barriers to approaching people from underserved communities can be achieved. The collaborative approach can lead to long-lasting change that genuinely makes a difference to patients' experience.^28^

However, patient participants from underserved communities may be more vulnerable and harder to reach or recruit than patients from the wider public who are not from these communities. We will ensure patients are fully informed about what is involved prior to the workshops. It is not anticipated that any participants will feel marginalised during the workshops as we will be guided by our PPI panel and use an ‘appreciative approach’ where all views will be valued. There will be sufficient facilitators during the workshops to accommodate participant needs. HW (Professor of e-Learning and Health Informatics and coinvestigator) has extensive experience in developing RLOs and will help facilitate the workshops.

#### Patient participation in interviews

Face-to-face interviews will take place in the patient's home, pharmacy or any other location according to the patient's wishes. A telephone interview (or Skype/FaceTime) option will also be available. Patients will receive at least 24 hours or as long as is needed to decide whether to take part in an interview. Interviews are not expected to raise any distress or make the patient upset as they will be centred on the patient's medicines, the levels of support they receive and how services such as the MUR can help them.

Should the researcher become aware that the patient requires urgent medical care, an intervention or is on the wrong medicines, they will be referred to their pharmacist, their GP or other health professional as appropriate.

### Dissemination

Following evaluation, the educational intervention will be made freely available online and accessible to pharmacy teams globally. The pharmacy workforce in the UK is made up of ∼50 000 registered pharmacists with 70% working within the community pharmacy sector.^29^ This study therefore has the potential to have an immediate beneficial impact for improving the delivery of MURs to underserved patients. The intervention will be available to the Centre for Pharmacy and Postgraduate Education (CPPE) and Higher Education Institutes (HEIs) that are responsible for pharmacist's MUR competency assessment and accreditation.

To support this study's contribution to wider knowledge, the research findings will be disseminated to regional, national and international audiences through conference and peer-reviewed publications targeted at service users, health professionals, academics, service commissioners and policymakers. We will also disseminate to pharmacy specific journals (eg, *The Pharmaceutical Journal* and *Chemist & Druggist*) and social media (eg, Twitter) to promote awareness.

## Conclusion

This paper is an important step in the dissemination process as it outlines the study's background, aims and details of methods that will be used. As well as providing pharmacy staff with knowledge and practical skills to engage patients from underserved communities, the study will also contribute to addressing the significant gap in the literature on how effective an intervention of this nature will be in improving pharmacy practice. In this respect, the study is novel and will provide information on the feasibility of the educational intervention, alongside the barriers and facilitators to implementation. Collectively, the findings from this study will act as the first stage in developing the coproduction methodology within community pharmacy and how this can be extended to address inequalities in other areas of pharmacy practice.
